# Beyond PD‐1: Mechanisms of Resistance to Checkpoint Blockade in Classical Hodgkin Lymphoma and Next‐Generation Immune Strategies

**DOI:** 10.1111/ejh.70101

**Published:** 2025-12-25

**Authors:** Santino Caserta, Enrica Antonia Martino, Mamdouh Skafi, Maria Eugenia Alvaro, Antonella Bruzzese, Nicola Amodio, Eugenio Lucia, Virginia Olivito, Caterina Labanca, Francesco Mendicino, Ernesto Vigna, Fortunato Morabito, Massimo Gentile

**Affiliations:** ^1^ Hematology Unit, Department of Onco‐Hematology AO of Cosenza Cosenza Italy; ^2^ Emergency and Internal Medicine Department Saint Joseph Hospital East Jerusalem Palestine; ^3^ Department of Experimental and Clinical Medicine University of Catanzaro Catanzaro Italy; ^4^ AIL Sezione di Cosenza Cosenza Italy; ^5^ Department of Pharmacy Health and Nutritional Science, University of Calabria Rende Italy

**Keywords:** classic Hodgkin lymphoma, ctDNA, PD‐1, PET

## Abstract

PD‐1 inhibitors have reshaped the treatment landscape of classical Hodgkin lymphoma, yet a substantial proportion of patients exhibit primary or acquired resistance driven by tumor‐intrinsic alterations, immunosuppressive microenvironmental signals, metabolic constraints, and EBV‐mediated modulation. This review summarizes key mechanisms underlying PD‐1 resistance and highlights emerging biomarkers—including early ^18^F‐FDG PET response, circulating tumor DNA kinetics, molecular subtyping, and spatial immune profiling—that enable early identification of nonresponders and support precision immunotherapy. Novel therapeutic strategies such as macrophage‐targeted agents, metabolic modulators, bispecific antibodies, low‐dose PD‐1 regimens, and CD30‐directed CAR‐T cells show promise in overcoming resistance, particularly when integrated into adaptive, biomarker‐guided treatment algorithms. Early incorporation of PET and ctDNA monitoring may inform timely treatment adaptation, while combination approaches addressing macrophage‐driven suppression or redundant immune checkpoints should be considered in biologically high‐risk patients. Overall, a deeper mechanistic understanding coupled with biomarker‐driven stratification is essential to optimize PD‐1‐based therapy and improve long‐term outcomes in cHL.

## 1 Introduction

Classical Hodgkin lymphoma (cHL) is a distinctive lymphoid malignancy characterized by rare neoplastic Hodgkin–Reed–Sternberg (HRS) cells embedded within a complex and immunologically active tumor microenvironment (TME) [1].

HRS cells frequently overexpress PD‐L1 and PD‐L2 due to 9p24.1 alterations and JAK2‐driven signaling, thereby promoting T‐cell exhaustion and immune evasion. These genomic and signaling abnormalities correlate with advanced‐stage disease and inferior progression‐free survival [2, 3].

The introduction of PD‐1 inhibitors, including nivolumab and pembrolizumab, has profoundly transformed the therapeutic landscape of cHL, yielding overall response rates (ORRs) of 65%–70% and durable complete responses (CRs) in approximately 20%–30% of heavily pretreated patients. Despite these substantial gains, up to one‐third of patients exhibit primary resistance, and an additional 30%–40% of initial responders eventually relapse, underscoring persistent biological barriers to long‐term disease control [4, 5, 6].

The recent adoption of nivolumab as part of frontline therapy in SWOG S1826 further amplifies the clinical relevance of PD‐1 resistance. Whereas immune checkpoint inhibitors were previously confined to the relapsed or refractory (R/R) setting—often in combination with GVD, ICE, or brentuximab vedotin—earlier exposure now raises critical questions regarding the optimal role of ICI‐based regimens in the second‐line setting, particularly for patients who experience early progression after frontline treatment. Resistance to PD‐1 blockade in cHL is multifactorial, arising from complex interactions between tumor‐intrinsic alterations, an immunosuppressive TME, metabolic constraints, Epstein–Barr virus (EBV)‐driven signaling, and host immune factors. These processes are frequently associated with high tumor burden, elevated inflammatory biomarkers, and recurrent genetic lesions [7, 8].

In this review, we integrate current mechanistic insights with emerging biomarkers and next‐generation immunotherapeutic strategies to elucidate how resistance to PD‐1 blockade develops, how it may be anticipated, and how therapeutic sequencing should adapt in the evolving era of frontline checkpoint inhibition.

## 2 Mechanisms of PD‐1 Resistance

PD‐1 resistance in cHL is multifactorial, reflecting the intricate interplay between malignant HRS cells and their immune microenvironment. Despite a histologically immune cell‐rich milieu, cHL paradoxically sustains profound immunosuppression, allowing HRS cell survival and expansion. Resistance to PD‐1 blockade arises through both extrinsic mechanisms, mediated by the TME, and intrinsic mechanisms encoded in the genetic, epigenetic, and metabolic circuits of HRS cells. Conceptually, these resistance pathways can be categorized into four overlapping domains: TME immunosuppression, metabolic reprogramming, HRS cell‐intrinsic alterations, and EBV‐mediated immune modulation.

### 2.1 TME Immunosuppression

The cHL TME represents one of the most striking examples of an immune cell‐rich malignancy. HRS cells comprise only 1%–2% of tumor mass, whereas the surrounding immune infiltrate—including T cells, B cells, natural killer (NK) cells, macrophages, dendritic cells, and stromal cells—accounts for 98%–99% [9, 10]. Paradoxically, this immunologically dense milieu fails to mount an effective antitumor response; instead, HRS cells actively reshape and co‐opt it into a functionally suppressive ecosystem that fosters immune tolerance and contributes directly to resistance to PD‐1 blockade [11].

HRS cells act as the “conductors” of the TME, orchestrating immune cell recruitment, differentiation, and polarization. Constitutive NF‐κB and JAK/STAT signaling drives secretion of chemokines such as CCL17 (TARC), CCL22, and CCL5, recruiting regulatory T cells (Tregs), myeloid‐derived suppressor cells (MDSCs), and M2‐polarized tumor‐associated macrophages (TAMs) [12]. M2‐polarized TAMs represent a central and recurrent immunosuppressive axis in cHL. These cells are actively recruited and functionally reprogrammed by HRS‐derived cytokines, reinforced by metabolic stress within the TME, and further amplified in EBV‐positive disease. Through the secretion of IL‐10, TGF‐β, VEGF, and arginase‐1, TAMs induce T‐cell dysfunction and exhaustion and are consistently associated with inferior response to PD‐1 blockade. HRS‐derived cytokines such as interleukin‐10 (IL‐10), transforming growth factor‐β (TGF‐β), and galectin‐1 reprogram recruited immune cells toward suppressive phenotypes, dampening cytotoxic T‐cell function [10, 13]. This dual “recruitment‐and‐education” strategy creates a protective niche resistant to PD‐1 blockade.

Tregs suppress CD8^+^ T‐cell cytotoxicity via IL‐10, TGF‐β, immune checkpoint expression (CTLA‐4 and LAG‐3), and cytotoxic granule release, and high Treg abundance correlates with poor PD‐1 responses [14, 15]. TAMs, generally M2‐skewed, secrete IL‐10, VEGF, and arginase‐1, suppressing T‐cell activity and correlating with inferior survival under PD‐1 therapy [1, 15, 16]. MDSCs contribute to resistance to PD‐1 blockade by depleting arginine and generating reactive oxygen and nitrogen species (ROS/RNS), thereby impairing T‐cell proliferation and effector function. In preclinical cHL models, pharmacologic inhibition of MDSCs using PDE5 inhibitors (e.g., sildenafil and tadalafil) or STAT3 inhibitors attenuated MDSC‐mediated immunosuppression, partially restored CD8^+^ T‐cell activity, and significantly enhanced the antitumor efficacy of PD‐1 blockade. Importantly, these effects were incomplete, and clinical validation in prospective human trials remains limited [17, 18, 19]. Stromal fibroblasts and mesenchymal cells further contribute to immune resistance in cHL by producing immunosuppressive mediators, including TGF‐β, CXCL12, and extracellular matrix components that limit T‐cell infiltration and promote myeloid cell dominance. Preclinical studies indicate that this stromal barrier can attenuate the efficacy of PD‐1 inhibition, highlighting a potential role for stromal remodeling and fibroblast‐targeting strategies as potential adjuncts to immunotherapy [20, 21].

### 2.2 Metabolic Barriers

Metabolic reprogramming within the cHL TME directly contributes to resistance to PD‐1 blockade. The high‐glycolytic activity of HRS cells limits glucose availability, thereby impairing CD8^+^ T‐cell proliferation, survival, and cytokine production. In parallel, indoleamine 2,3‐dioxygenase (IDO)‐mediated tryptophan depletion and arginase‐1‐dependent arginine consumption promote T‐cell dysfunction and favor the expansion of regulatory T cells. Hypoxia‐driven HIF‐1α signaling further enhances PD‐L1 expression and extracellular adenosine accumulation through the CD39/CD73 axis, resulting in additional suppression of both T and NK cell activity [22, 23, 24].

In preclinical lymphoma models, pharmacologic targeting of IDO or adenosine signaling pathways partially restores effector T‐cell function and enhances the antitumor activity of PD‐1 blockade. Although these findings support metabolic modulation as a rational combinatorial strategy, clinical validation remains limited and warrants further investigation in prospective studies [25, 26, 27, 28].

### 2.3 HRS Cell‐Intrinsic Alterations

Intrinsic resistance to PD‐1 blockade in cHL is largely driven by genetic, epigenetic, and signaling abnormalities of HRS cells that collectively blunt immune recognition and sustain immune evasion despite checkpoint inhibition. A central mechanism involves defective antigen presentation. Loss‐of‐function mutations or deletions of β_2_‐microglobulin (B2M), together with recurrent alterations in Class II major histocompatibility complex (MHC) transactivator (CIITA), result in reduced or absent expression of MHC Class I and Class II molecules, respectively. This dual impairment compromises both CD8^+^ cytotoxic T‐cell recognition and CD4^+^ T‐cell help, effectively rendering HRS cells immunologically “invisible” and reducing tumor dependence on PD‐1–PD‐L1 interactions for immune escape [3, 29, 30].

Beyond impaired antigen presentation, HRS cells frequently engage redundant inhibitory pathways that bypass PD‐1 blockade. Expression of alternative immune checkpoint ligands—including galectin‐9 (ligand for TIM‐3), CD155 (ligand for TIGIT), and, in some cases, HVEM interactions—provides parallel suppressive signals that maintain T‐cell exhaustion. These compensatory checkpoints are often co‐expressed in PD‐1‐refractory disease and correlate with transcriptional programs of terminal T‐cell dysfunction, offering a mechanistic explanation for why clinical failure of PD‐1 monotherapy may occur despite adequate target engagement [20].

Constitutive oncogenic signaling further reinforces intrinsic resistance. Aberrant activation of the JAK–STAT pathway—driven by 9p24.1 amplification, JAK2 copy number gains, or activating STAT mutations—supports HRS cell survival while directly upregulating PD‐L1/PD‐L2, and other immunomodulatory genes. In parallel, chronic NF‐κB activation, sustained by mutations in negative regulators (e.g., TNFAIP3) or by persistent receptor‐mediated signaling, promotes proliferation, resistance to apoptosis, and secretion of cytokines that shape an immunosuppressive microenvironment. Importantly, these oncogenic pathways remain active even in the presence of PD‐1 blockade, enabling tumor persistence and disease progression [31, 32].

Epigenetic reprogramming provides an additional layer of stability to these immune escape mechanisms. DNA hypermethylation and histone modifications silence genes involved in antigen processing, interferon signaling, and immune synapse formation, thereby locking HRS cells into a dedifferentiated, immune‐resistant transcriptional state. This epigenetic rigidity limits the capacity of immune pressure induced by PD‐1 blockade to reprogram tumor‐cell behavior and restore effective immune recognition.

From a therapeutic perspective, multiple strategies are being explored to counteract HRS cell‐intrinsic resistance. Epigenetic modifiers, including histone deacetylase and DNA methyltransferase inhibitors, can partially restore MHC expression and interferon responsiveness, thereby increasing tumor immunogenicity. In parallel, combinatorial approaches targeting compensatory immune checkpoints, such as TIM‐3, TIGIT, or LAG‐3, aim to dismantle redundant inhibitory circuits that sustain T‐cell exhaustion. Finally, pharmacologic inhibition of JAK–STAT or NF‐κB signaling is under investigation to simultaneously weaken tumor survival programs and attenuate tumor‐driven immune suppression, offering a rational strategy to re‐sensitize intrinsically resistant cHL to immune‐mediated control [33, 34, 35].

### 2.4 EBV‐Mediated Modulation

EBV infection is observed in a substantial proportion of cHL cases—approximately 30%–50% of adult cases and higher in many pediatric cohorts—and substantially influences both tumor‐cell intrinsic and microenvironmental pathways of immune evasion [15]. HRS cells in EBV‐positive disease express latent viral proteins such as latent‐antigen LMP1 and LMP2A/B, which mimic constitutive CD40 and B‐cell receptor (BCR) signaling, respectively, thereby driving NF‐κB and JAK/STAT activation, antiapoptotic gene expression, and immune checkpoint ligand upregulation [25]. In particular, LMP1 has been shown to induce PD‐L1 expression via STAT signaling in Hodgkin lymphoma and other EBV‐associated lymphomas.

While EBV‐positive cHL is characterized by viral antigen expression and enhanced PD‐L1 induction—features that could theoretically increase sensitivity to immune‐based therapies—clinical outcomes with PD‐1 blockade remain inconsistent. Some studies report comparable or even higher initial response rates in EBV‐positive disease, whereas others show no significant differences in progression‐free or overall survival compared with EBV‐negative cases. This apparent discrepancy likely reflects the dual role of EBV, which simultaneously increases tumor immunogenicity while reinforcing immune evasion through IL‐10 and TGF‐β secretion, expansion of regulatory T cells, and polarization of M2 macrophages, ultimately promoting adaptive resistance.

In addition to these intrinsic tumor‐cell effects, EBV drives microenvironmental immune modulation. EBV‐encoded latent proteins and viral microRNAs promote secretion of immunosuppressive cytokines (e.g., IL‐10 and TGF‐β) and recruit Tregs, creating a TME that is uniquely favorable for immune escape [35]. This combined effect of HRS intrinsic changes plus EBV‐driven TME remodeling may contribute both to the observed initial responsiveness and later adaptive resistance to PD‐1 blockade in EBV‐positive cHL.

EBV‐positive cHL exhibits distinct biological features; however, its impact on PD‐1 response remains uncertain. Some studies suggest similar or slightly enhanced responsiveness due to high PD‐L1 induction, while others show no outcome differences.

Therapeutically, EBV‐directed strategies—such as EBV‐specific cytotoxic T‐cell infusions, LMP1/LMP2 peptide vaccines, and adoptive immunotherapies—have been evaluated in EBV‐associated lymphomas and may hold particular promise when combined with checkpoint inhibition to overcome resistance that is both intrinsic and microenvironmental [36].

### 2.5 Other Strategies to Enhance PD‐1 Blockade Efficacy

Other emerging therapeutic strategies in cHL involve bispecific antibodies (BsAbs) and bifunctional fusion proteins, which are designed to simultaneously engage tumor antigens and immune effector pathways or modulate the immunosuppressive TME. These approaches aim to overcome resistance mechanisms and enhance antitumor immunity, particularly in R/R disease.

AFM13 is among the most extensively studied BsAbs in cHL. It targets CD30 on HRS cells and CD16A on NK cells, promoting antibody‐dependent cellular cytotoxicity (ADCC). In a Phase 1b trial, patients with R/R cHL treated with AFM13 plus pembrolizumab achieved an ORR of 88% and a CR rate of 46%, including those previously exposed to PD‐1 inhibitors. The combination demonstrated a manageable safety profile, with treatment‐related adverse events primarily consisting of Grade 1–2 infusion reactions and cytokine release syndrome (CRS) [37].

BsAbs targeting immune checkpoint and innate resistance pathways are also under investigation. IBI322, a CD47/PD‐L1 BsAb, simultaneously blocks CD47‐mediated phagocytosis resistance and PD‐L1‐driven T‐cell suppression. Early‐phase data in patients with PD‐1 or PD‐L1–refractory cHL suggest favorable safety and preliminary clinical activity, although detailed efficacy results remain unpublished [38].

Next‐generation BsAbs directed against CD123, expressed on HRS cells and TME components, are in development. MGD024, a CD123 × CD3 molecule, redirects cytotoxic T cells toward CD123‐positive cells while limiting CRS through optimized binding and controlled T‐cell activation. In the ongoing first‐in‐human Phase I trial (NCT05362773) enrolling R/R hematologic malignancies, MGD024 has shown an acceptable safety profile and early signs of efficacy, though cHL‐specific outcomes are not yet reported (NCT05362773).

Bifunctional fusion proteins offer an additional layer of immunomodulation. SHR‐1701, combining PD‐L1 blockade with TGF‐β neutralization, targets both inhibitory checkpoints and the immunosuppressive TME. Initially explored in solid tumors, recent Phase I data in heavily pretreated cHL patients treated with SHR‐1701 plus the enhancer of zeste homolog 2 (EZH2) inhibitor SHR2554 (NCT04407741) showed an ORR of 100%, with complete remission in 7% of patients and an acceptable safety profile. These results support further investigation of SHR‐1701‐based regimens in R/R cHL [NCT04407741].

Collectively, these innovative therapies—BsAbs and bifunctional fusion proteins—represent complementary strategies to restore antitumor immunity in cHL. By engaging both tumor cells and immune effectors while modulating the TME, they offer potential to overcome intrinsic resistance and enhance the efficacy of existing immunotherapies.

Low‐dose or metronomic PD‐1 blockade may further enhance efficacy while reducing immune‐related toxicity by recalibrating T‐cell activation thresholds and promoting memory formation. Chan et al. [39] report that low‐dose pembrolizumab and nivolumab are both effective and well tolerated in patients with R/R classical cHL treated in a resource‐constrained setting. Mechanistically, low‐dose therapy may promote T‐cell memory and prevent overstimulation, potentially enhancing the durability of response. Clinically, this approach offers a feasible option for patients with comorbidities, prior therapy exposure, or restricted access to full‐dose immunotherapy. Future studies should refine the optimal minimum effective dose and explore integration with complementary immunomodulatory strategies—such as metabolic modulators, TAM‐directed therapies, BsAbs, or chimeric antigen receptor (CAR‐T) cells—to maximize efficacy while minimizing toxicity. Early‐phase trials are evaluating these approaches in combination with TAM modulators, metabolic therapies, or BsAbs.

CAR‐T‐cell therapy targeting CD30 or EBV antigens complements checkpoint blockade, particularly in PD‐1‐resistant cHL. CAR‐T cells bypass HLA‐dependent antigen presentation, and their combination with metabolic modulators or TAM‐directed therapies may improve persistence and cytotoxicity within the hostile TME. However, experience with CD30‐directed T‐cell‐engaging strategies has highlighted important biological and clinical limitations that must be carefully considered when positioning these approaches as future therapeutic directions. CD30 × CD3 BsAbs and CD30‐directed CAR‐T cells have demonstrated proof‐of‐principle activity in R/R cHL, yet ORRs have been modest and responses often short‐lived across multiple trials, particularly outside highly selected patient populations. A major biological challenge lies in the heterogeneous and frequently low‐density expression of CD30 on HRS cells, coupled with antigen shedding and trogocytosis, which may facilitate immune escape and contribute to early relapse.

Toxicity has also emerged as a critical concern. CD30 is not strictly tumor‐specific and is expressed on activated T cells and subsets of normal immune cells, predisposing to on‐target, off‐tumor effects. In CD30‐directed CAR‐T‐cell studies, CRS, prolonged cytopenias, and T‐cell fratricide have been reported, while CD30 × CD3 BsAbs carry a risk of excessive T‐cell activation, immune exhaustion, and cumulative toxicity with repeated dosing. Moreover, the profoundly immunosuppressive cHL microenvironment—characterized by abundant M2‐polarized macrophages, regulatory T cells, and metabolic constraints—further limits CAR‐T‐cell persistence and effector function.

Collectively, these observations suggest that CD30‐directed cellular therapies are unlikely to be sufficient as standalone approaches and will instead require rational optimization. Potential approaches include improved antigen‐binding and signaling design, incorporation of safety switches, combination with checkpoint blockade or TAM‐reprogramming agents, and careful patient selection. Thus, while CD30 remains an attractive therapeutic target, its successful exploitation will depend on overcoming both antigen‐related limitations and microenvironment‐driven resistance [40].

Rational sequencing strategies integrate these modalities into a coherent therapeutic continuum: the TME is first conditioned through TAM‐directed or metabolic interventions to create a more immunopermissive niche. On this primed landscape, PD‐1 blockade serves to reinvigorate exhausted T cells, while BsAbs or CAR‐T cells add a layer of highly targeted cytotoxicity.

Throughout this process, adaptive clinical decision‐making is enabled by dynamic monitoring with circulating tumor DNA (ctDNA), immune‐gene expression signatures, and positron–emission tomography (PET) imaging, which refine both treatment intensity and temporal sequencing. Such mechanism‐driven, biomarker‐guided orchestration addresses metabolic, microenvironmental, and HRS cell‐intrinsic barriers in concert, ultimately enhancing the precision, durability, and overall efficacy of PD‐1‐based immunotherapy in cHL.

**FIGURE 1:**
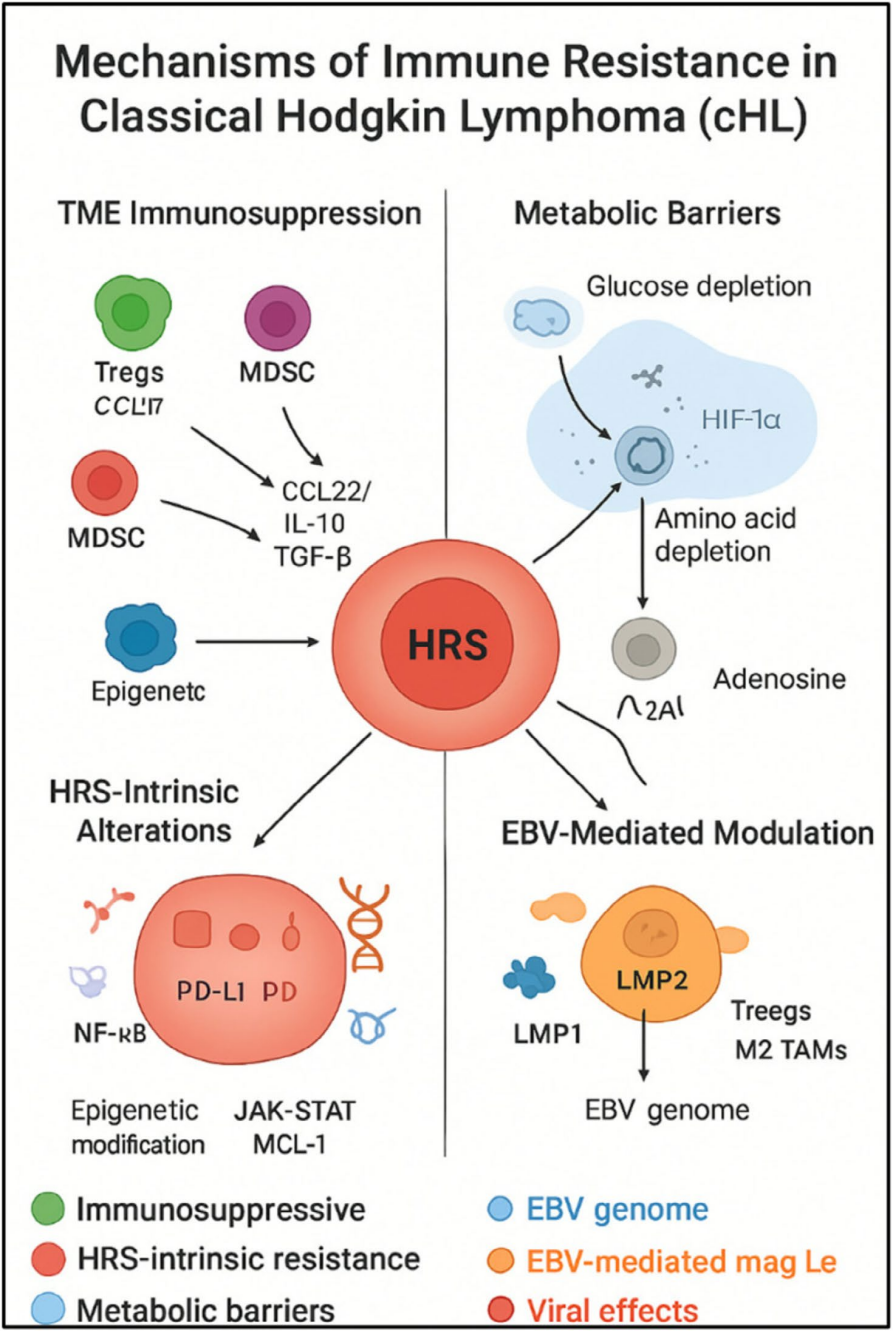
Mechanisms of PD‐1 resistance in classical Hodgkin lymphoma (cHL). PD‐1 resistance in cHL results from both tumor‐intrinsic and microenvironmental factors. The TME contains immunosuppressive cells (Tregs, TAMs, MDSCs) that inhibit cytotoxic T cells through cytokines and alternative checkpoints. Metabolic constraints such as nutrient depletion, hypoxia, and adenosine accumulation further impair T‐cell function. HRS cells exhibit intrinsic alterations, including loss of HLA molecules, constitutive NF‐κB/JAK–STAT signaling, and epigenetic changes that reduce antigen presentation. EBV infection contributes to enhancing PD‐L1 expression and reshaping the TME. Together, these interconnected pathways define potential targets for combination strategies to overcome PD‐1 resistance.

Figure 1 summarizes the interconnected mechanisms underlying resistance to PD‐1 in cHL. Although resistance is commonly described across four domains—TME‐mediated immunosuppression, metabolic constraints, HRS cell‐intrinsic alterations, and EBV‐driven modulation—these processes function as an integrated and self‐reinforcing network rather than as independent pathways. Tumor‐intrinsic alterations such as 9p24.1 amplification, JAK/STAT and NF‐κB activation, and loss of antigen presentation promote the secretion of immunosuppressive cytokines and chemokines that recruit Tregs, M2 macrophages, and MDSCs. These immune populations, in turn, reinforce tumor‐intrinsic escape programs by releasing IL‐10, TGF‐β, VEGF, ROS/RNS, and alternative checkpoint ligands, ultimately sustaining T‐cell exhaustion even in the presence of PD‐1 blockade.

Metabolic barriers, including glucose competition, amino acid depletion, hypoxia, and extracellular adenosine accumulation, intersect with both TME suppression and tumor‐intrinsic signaling by impairing T‐cell fitness, promoting macrophage polarization, and enhancing PD‐L1 expression. When present, EBV infection further amplifies each of these axes through LMP1‐ and LMP2‐mediated activation of NF‐κB and JAK/STAT pathways, as well as through cytokine‐driven recruitment of Treg and M2 macrophages. Collectively, these convergent mechanisms establish a resilient immune‐evasive ecosystem that limits the durability of PD‐1‐mediated tumor control.

## 3 Biomarkers Predicting Resistance

To improve clarity and coherence, predictive biomarkers of resistance to PD‐1 blockade can be conceptually organized into distinct yet complementary domains, including functional imaging, ctDNA, tumor genomic alterations, and TME‐derived immune signatures. This framework mirrors the mechanistic architecture described in the resistance section and enables a more systematic interpretation of available evidence.

Functional imaging provides a genuinely noninvasive window into TME activity and early determinants of therapeutic outcome. PET, whether employing conventional ^18^F‐fluorodeoxyglucose (^18^F‐FDG) or emerging immuno/metabolic tracers, quantifies tumor metabolic burden, spatial heterogeneity, and features that infer immune‐cell activity, thereby capturing early functional changes during PD‐1‐directed therapy. In this setting, early ^18^F‐FDG PET/CT responses have been correlated with clinical outcomes in Hodgkin lymphoma treated with PD‐1 inhibitors, and PET metrics can identify early signals of treatment failure—for example, persistent hypermetabolism or heterogeneous uptake patterns that may reflect immune escape rather than true treatment effect [41, 42].

Volumetric and radiomic PET features—quantitative features (spatial, textural, morphological) from PET/CT images useful to capture tumor heterogeneity and biological characteristics—are increasingly conceptualized as “immunometabolic” scores that reflect both tumor metabolic activity and indirect signals of tumor–immune interactions. Radiomic analyses capture spatial and textural features invisible to conventional visual interpretation, enabling quantification of tumor heterogeneity and early immunological dynamics. In solid tumors, such as non–small cell lung cancer, PET radiomics has been shown to predict benefit from checkpoint inhibitors, illustrating the principle that image‐derived quantitative features can serve as surrogates for immune biology [43, 44].

Importantly, the integration of PET biomarkers with molecular and transcriptomic data improves predictive accuracy. Combining immunometabolic scores with PD‐L1/PD‐L2 expression, immune‐gene signatures, and ctDNA kinetics provides a more comprehensive portrait of the tumor–immune interface. Such integration allows identification of patients at risk of early treatment failure and supports adaptive, biomarker‐driven modification of therapy even before clinical progression becomes apparent [44]. In R/R cHL, early PET responses during PD‐1 blockade strongly correlate with progression‐free survival, highlighting the prognostic value of metabolic imaging as a surrogate for therapeutic efficacy [25].

Recent advances in high‐throughput sequencing and liquid biopsy technologies have profoundly expanded our capacity to dissect the genomic and immunologic complexity of cHL, revealing that resistance to immunotherapy is not a uniform process but the result of multifaceted TME interactions. Historically, the study of cHL genomics was limited by the scarcity of malignant HRS cells within tumor biopsies, making comprehensive tissue‐based sequencing technically challenging. This limitation has been overcome by the advent of ctDNA profiling, which allows for noninvasive, sensitive, and longitudinal analysis of tumor‐derived genetic material in plasma.

Among the most transformative contributions in this field is the study by Alig et al., which provided a panoramic view of the cHL genomic landscape through ultra‐deep hybrid‐capture sequencing of ctDNA in more than 400 patients across international centers [45]. This work validated ctDNA as a robust surrogate for tissue biopsies, demonstrating near‐perfect concordance between plasma‐ and tissue‐derived mutation profiles. The authors identified four molecular subtypes of cHL, each defined by unique genetic and immunologic features: a B2M‐deficient subtype, associated with defective antigen presentation and immune escape; an NF‐κB‐driven subtype, enriched in mutations that activate inflammatory survival pathways; a JAK/STAT subtype, typical of nodular sclerosis histology; and a hypermutated or apolipoprotein B mRNA editing enzyme, catalytic polypeptide‐like (APOBEC)‐high group, indicative of defective DNA repair mechanisms.

The biological and clinical implications of this genomic taxonomy extend beyond descriptive classification. Although the four molecular subtypes differ in EBV association, clinical stage, and progression‐free survival, their capacity to inform therapeutic decision‐making is only beginning to be defined. B2M‐deficient and APOBEC‐high tumors, characterized by impaired antigen presentation or genomic instability, are associated with inferior outcomes and may require combination strategies aimed at enhancing tumor immunogenicity or targeting alternative immune checkpoints. In contrast, NF‐κB‐activated and JAK/STAT‐driven subtypes may be particularly amenable to pathway‐specific inhibitors integrated with PD‐1 blockade. Importantly, ctDNA burden and clonal diversity correlate with therapeutic response across subtypes, supporting ctDNA as a dynamic biomarker to track disease biology, detect early resistance, and potentially guide subtype‐adapted therapeutic strategies [46].

Complementary studies have highlighted the role of recurrent copy number variations (CNVs) detectable in ctDNA. Gains of 9p24.1 (PD‐L1/PD‐L2), REL/JAK2 amplifications, and losses of B2M/CIITA are recurrent events linked to tumor aggressiveness, metabolic activity, and immune evasion [47]. Serial monitoring of ctDNA allows real‐time tracking of clonal evolution, detection of emerging resistant clones, and anticipation of radiographic relapse [48]. Similarly, in Chinese patients treated with sintilimab, baseline ctDNA levels and the magnitude of its decline correlated with clinical response, underscoring the value of ctDNA as a predictive and pharmacodynamic biomarker.

In addition to ctDNA, the TME is a major determinant of PD‐1 inhibitor efficacy. High‐dimensional transcriptomic analyses, including spatial transcriptomics, reveal that TME composition and functional orientation correlate strongly with treatment outcomes. Enrichment of immunosuppressive signatures, such as regulatory T cells, M2 macrophages, and inhibitory checkpoint ligands including LAG‐3 and TIM‐3, is associated with primary resistance. Conversely, inflamed TMEs rich in cytotoxic T cells predict favorable responses. Spatial mapping of immune populations within the TME has shown that the proximity of effector T cells to HRS cells and the presence of immunosuppressive niches can critically influence PD‐1 blockade efficacy [9].

Because resistance mechanisms in cHL are multifactorial, integrative strategies combining ctDNA kinetics, immune‐gene expression, spatial TME profiling, and functional imaging provide superior predictive power. High‐risk profiles—characterized by persistent ctDNA, suppressive TME signatures, and unfavorable PET‐immunometabolic scores—may benefit from combination therapies, including PD‐1 inhibitors with macrophage reprogramming agents or BsAbs. Conversely, patients with rapid ctDNA clearance, an inflamed TME, and favorable PET responses are optimal candidates for PD‐1 monotherapy or therapy de‐escalation. Such composite biomarker frameworks enable adaptive, precision immunotherapy, facilitating early intervention and treatment personalization based on biological signals rather than conventional clinical endpoints.

The integration of PET radiomics, ctDNA, and immune landscape characterization represents a paradigm shift in cHL management. These tools enable early identification of patients at risk for PD‐1 resistance, rational selection of combination or sequential therapies, adaptive trial designs guided by mechanistic biomarkers, and precision medicine approaches to maximize response durability. Addressing PD‐1 resistance in cHL requires multifactorial strategies targeting tumor‐intrinsic, microenvironmental, metabolic, and viral mechanisms. Rational combination and sequencing approaches aim to synergize PD‐1 blockade with complementary interventions, ultimately enhancing response rates and long‐term therapeutic durability.

Despite promising early evidence, the use of ctDNA and PET to guide adaptive therapy in cHL requires prospective validation. Key unmet needs include the definition of standardized ctDNA thresholds and sampling schedules; the identification of PET‐derived metrics and optimal timepoints that reliably distinguish true resistance from immune‐related or inflammatory effects; and demonstration that biomarker‐triggered treatment modifications improve clinical outcomes in randomized or adaptive trial designs. In addition, multicenter reproducibility, cost‐effectiveness, and applicability across diverse patient subgroups must be established before these approaches can be implemented into routine clinical practice.

**FIGURE 2:**
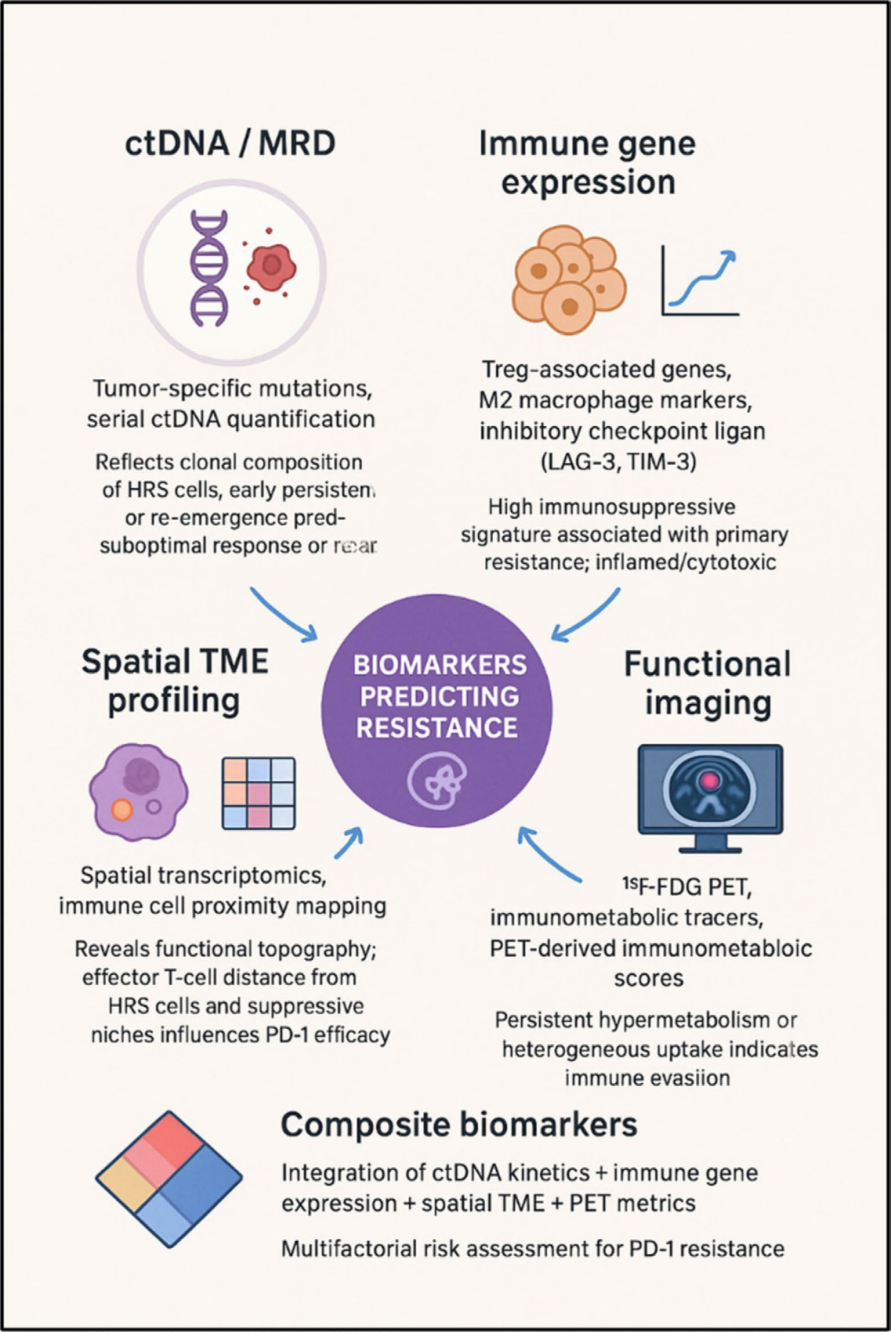
Predictive biomarkers of PD‐1 resistance in classical Hodgkin lymphoma (cHL). Key biomarker categories include circulating tumor DNA (ctDNA) and MRD monitoring for early relapse detection, immune‐gene expression profiling to distinguish suppressive versus inflamed TMEs, spatial mapping to assess effector–suppressor interactions, and functional imaging (e.g., ^18^F‐FDG PET) to reveal early resistance. Integrating these modalities enables multimodal risk stratification and precision immunotherapy in cHL.

Figure 2 summarizes the predictive biomarkers of PD‐1 resistance in cHL.

## 4 Conclusions

PD‐1 blockade has profoundly transformed the therapeutic landscape of cHL, achieving unprecedented response rates and durable remissions, even among heavily pretreated patients. However, a considerable subset of patients experiences primary or acquired resistance, which limits the long‐term efficacy of immune checkpoint inhibitors. This resistance is multifactorial, encompassing tumor‐intrinsic genomic alterations, immunosuppressive features of the TME, metabolic constraints, and EBV‐driven immune modulation—all converging to impair T‐cell function and immune surveillance.

Advances in mechanistic understanding have fostered the development of rational, next‐generation therapeutic strategies. The integration of predictive biomarkers—such as ctDNA, MRD kinetics, immune‐gene expression profiles, spatial TME mapping, molecular subtyping, and functional PET imaging—enables the early identification of patients at elevated risk of therapeutic failure. These biomarkers offer both prognostic and pharmacodynamic insights, facilitating adaptive, patient‐tailored interventions that address the dominant mechanisms of resistance.

Emerging combinatorial and sequential approaches—including metabolic modulators, TAM‐targeted agents, BsAbs, low‐dose PD‐1 regimens, and adoptive cellular therapies such as CAR‐T cells—represent a multipronged strategy to overcome resistance. By reprogramming the immunosuppressive TME, restoring T‐cell fitness, counteracting HRS cell‐intrinsic immune evasion, and enhancing tumor‐directed cytotoxicity, these interventions aim to deepen and prolong clinical responses.

Looking ahead, adaptive, biomarker‐driven clinical trials will be crucial to translating these mechanistic insights into practice. Real‐time monitoring of ctDNA/MRD dynamics, immune signatures, and functional imaging parameters can support early therapeutic adaptation, guide combination strategies, and validate biomarkers as decision tools. This evolution marks a paradigm shift from empiric PD‐1 blockade toward precision, mechanism‐informed immunotherapy, expanding the population that benefits from checkpoint inhibition while minimizing unnecessary toxicity.

**FIGURE 3:**
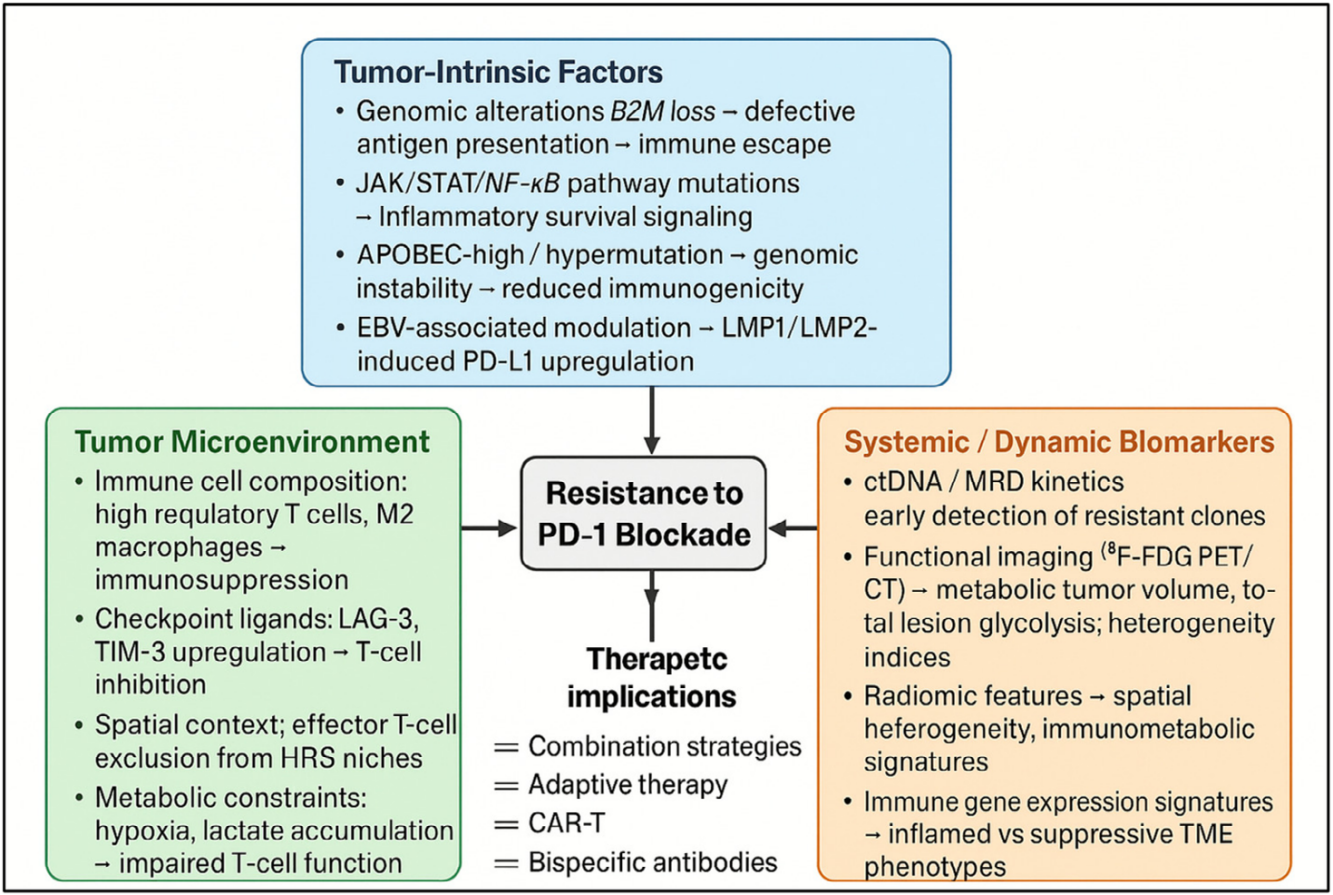
Mechanisms of resistance to PD‐1 blockade in classical Hodgkin lymphoma (cHL). Resistance arises from tumor‐intrinsic, microenvironmental, and systemic factors. Tumor‐intrinsic mechanisms include B2M loss, JAK/STAT/NF‐κB mutations, APOBEC‐driven hypermutation, and EBV‐induced PD‐L1 upregulation, leading to immune escape. The tumor microenvironment contributes through regulatory T cells, M2 macrophages, LAG‐3/TIM‐3 upregulation, and metabolic constraints such as hypoxia. Systemic biomarkers help identify resistant phenotypes. These insights support the development of combination therapies, adaptive immunotherapy, and bispecific antibodies to overcome resistance.

In summary (Figure 3), the convergence of mechanistic biology, predictive biomarkers, and rational combination therapies defines the next frontier in cHL immunotherapy. Sustained translational research and innovative clinical trial designs are pivotal to overcoming PD‐1 resistance, personalizing therapy, and ultimately achieving durable, potentially curative outcomes for patients with cHL.

## Author Contributions

Conceptualization: Santino Caserta, Enrica Antonia Martino, Mamdouh Skafi, Fortunato Morabito, and Massimo Gentile. Methodology: Enrica Antonia Martino, Francesco Mendicino, Ernesto Vigna, Antonella Bruzzese, and Fortunato Morabito. Writing – original draft preparation: Enrica Antonia Martino, Santino Caserta, Fortunato Morabito, and Massimo Gentile. Writing – review and editing: Enrica Antonia Martino, Santino Caserta, Mamdouh Skafi, Fortunato Morabito, and Massimo Gentile. All authors have read and agreed to the published version of the manuscript.

## Funding

The authors have nothing to report.

## Ethics Statement

The authors have nothing to report.

## Conflicts of Interest

The authors declare no conflicts of interest.

## Data Availability

Data sharing not applicable to this article as no datasets were generated or analysed during the current study.
